# Supplementary material for: Identification of photoperiod-induced specific miRNAs in the adrenal glands of Sunite sheep (Ovis aries)
Source: Front Vet Sci. 2022 Jul 22;9:888207. doi: 10.3389/fvets.2022.888207 (PMC9354845; doi:10.3389/fvets.2022.888207)
Supplement: Supplementary file 1 [file Data_Sheet_1.ZIP › Supplementary file/miRDeep2 file.zip!/miRDeep2 file/Know_Structure/LP42A2/oar-let-7g.pdf]

miRBase precursor : oar-let-7g
Total read count : 642521
oar-let-7g read count : 642502
remaining reads : 19

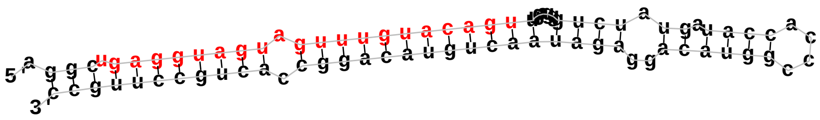

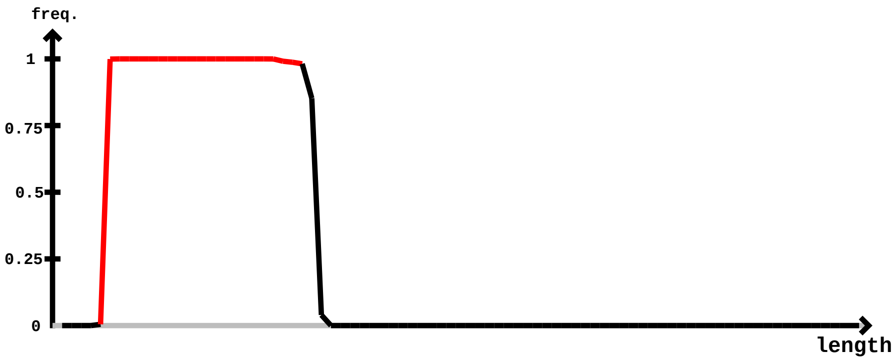

oar-let-7g

```
5'- aggcugagguaguaguuuguacaguuugagggucuaugauaccacccgguacaggagauaacuguacaggccacugccuugcc   -3'   exp
    .(((.((((((((.((((((((((((.....((((.((.((((....))))))..)))))))))))))))).)))))))))))   reads   mm   sample
    ...cugagguaguaguuugua..............................................................   1       0    seq
    ...cugagguaguaguuuguac.............................................................   39      0    seq
    ...cugagguaguaguuuguaca............................................................   15      0    seq
    ...cugagguaguaguuuguacag...........................................................   28      0    seq
    ...cugagguaguaguuuguacagu..........................................................   603     0    seq
    ...cugagguaguaguuuguacaguu.........................................................   2312    0    seq
    ...cugagguaguaguuuguacaguuu........................................................   109     0    seq
    ....ugUgguaguaguuugua..............................................................   3       1    seq
    ....ugaAguaguaguuugua..............................................................   1       1    seq
    ....ugagguaguaguuuAua..............................................................   1       1    seq
    ....ugagguaguaguuugua..............................................................   169     0    seq
    ....ugagguagCaguuuguac.............................................................   10      1    seq
    ....ugagguaguaguuuguac.............................................................   5434    0    seq
    ....ugagguaguaguuuguaca............................................................   2493    0    seq
    ....ugagguaguaguuuguacag...........................................................   3653    0    seq
    ....ugagguaguaguuuguacagu..........................................................   82295   0    seq
    ....ugagguaguaguuuguacaguu.........................................................   519497  0    seq
    ....ugagguaguaguuuguacaguuu........................................................   25373   0    seq
    ....ugagguaguaguuuguacaguuug.......................................................   21      0    seq
    ....ugagguaguaguuuguacaguuuga......................................................   3       0    seq
    .....gagguaguaguuuguacagu..........................................................   59      0    seq
    .....gagguaguaguuuguacaguu.........................................................   357     0    seq
    .....gagguaguaguuuguacaguuu........................................................   20      0    seq
    ......agguaguaguuuguacaguu.........................................................   5       0    seq
    .......gguaguaguuuguacaguu.........................................................   1       0    seq
    ..........................................................uaacuguacaggccacugccuugc.   1       0    seq
    ............................................................acuguacaggccacugccu....   1       0    seq
    ............................................................acuguacaggccacugccuugc.   2       0    seq
    .............................................................cuguacaggccacugccuugc.   12      0    seq
    .............................................................cuguacaggccacugccuugcc   3       0    seq
```
